# Molecular Typing of Protease-Resistant Prion Protein in Transmissible Spongiform Encephalopathies of Small Ruminants, France, 2002–2009

**DOI:** 10.3201/eid1701.100891

**Published:** 2011-01

**Authors:** Johann Vulin, Anne-Gaëlle Biacabe, Géraldine Cazeau, Didier Calavas, Thierry Baron

**Affiliations:** Author affiliation: Agence Nationale de Sécurité Sanitaire, Lyon, France

**Keywords:** Prion, BSE, TSE, CH1641, scrapie, sheep, goat, molecular typing, France, research

## Abstract

The agent that causes bovine spongiform encephalopathy (BSE) may be infecting small ruminants, which could have serious implications for human health. To distinguish BSE from scrapie and to examine the molecular characteristics of the protease-resistant prion protein (PrP^res^), we used a specifically designed Western blot method to test isolates from 648 sheep and 53 goats. During 2002–2009, classical non-Nor98 transmissible spongiform encephalopathy had been confirmed among ≈1.7 million small ruminants in France. Five sheep and 2 goats that showed a PrP^res^ pattern consistent with BSE, or with the CH1641 experimental scrapie source, were identified. Later, bioassays confirmed infection by the BSE agent in 1 of the 2 goats. Western blot testing of the 6 other isolates showed an additional C-terminally cleaved PrP^res^ product, with an unglycosylated band at ≈14 kDa, similar to that found in the CH1641 experimental scrapie isolate and different from the BSE isolate.

Transmissible spongiform encephalopathies (TSEs) are a group of fatal neurodegenerative diseases that include scrapie in sheep and goats, bovine spongiform encephalopathy (BSE) in cattle, and Creutzfeldt-Jakob disease (CJD) in humans (*1**,**2*). TSEs are characterized by accumulation in the brain of a disease-associated isoform (PrP^d^) of a host-encoded cellular prion protein (PrP^c^) (*3*). PrP^d^, in comparison with the normal prion protein PrP^c^, clearly differs in secondary and tertiary structures (*4**,**5*) and in biochemical characteristics (*6*). Proteinase K (PK) digestion destroys PrP^c^, but in PrP^d^ it generates a protease-resistant fragment known as PrP^res^. Most TSE diagnostic methods (e.g., ELISA and Western blot tests) are based on detection of PrP^res^ (*7*).

The transmissible agent involved in BSE in cattle is known to cause prion diseases in other species under natural conditions (*8*). BSE can also be experimentally transmitted to sheep and goats, including after oral challenge to test for transmission (*9*). Because BSE-contaminated meat and bone meal may have been fed to small ruminants, BSE may have been transmitted to sheep or goats. Also, the Scientific Steering Committee of the European Commission has hypothesized that the BSE agent might have originated from a scrapie agent in sheep or goats and that these animals may represent a reservoir (*10*). In view of these data, the European Commission defined a strategy to investigate the possible presence of BSE in sheep and goats under natural conditions (*11*).

The standard for strain typing TSE agents is based on analysis of the phenotypic characteristics of the disease after transmission in laboratory rodents. Biological characterization of the BSE agent in inbred wild-type mice appeared to be reliable, because it showed uniform features in mice (*8*). However, this approach is time-consuming and costly. The identification of uniform molecular features of PrP^res^ by Western blot in human variant CJD paved the way to a similar approach for detecting possible BSE in small ruminants (*12*). The molecular criteria defined in these studies included electrophoretic mobilities, glycosylation characteristics, and immunolabeling with different monoclonal antibodies (*13*). The last criteria enabled mapping of the protease cleavage site of the PrP protein fragment obtained after PK digestion (*14*). More recently, the identification of additional C-terminal PrP^res^ products may contribute to discrimination of the different types of CJD (*15*) or of different scrapie and BSE sources (*16**,**17*). Discriminant molecular features of the prion protein can also be investigated by immunohistochemical analysis (*18*) or ELISA (*19*). In all of these studies, it was assumed that the strain information was closely associated with the structural features of PrP^d^.

The Western blot method enabled discrimination of experimental BSE in sheep from most scrapie-affected animals (*12**,**13**,**20**–**24*). Nevertheless, discrimination was more difficult with the CH1641 experimental scrapie isolate (*21**,**25*), which otherwise clearly differs from BSE by its absence of transmissibility to wild-type mice (*26*). Similar molecular features to those of CH1641 have been described in a few natural scrapie cases in France (*27*) and in the United Kingdom (*24*). We describe the molecular findings obtained for a large series of TSE infections in France identified in small ruminants by active surveillance during 2002–2009 and for CH1641-like isolates in sheep and in 1 goat.

## Materials and Methods

### Animals and Tissues

Two samples of BSE in small ruminants were used as controls, 1 (SB1) from a sheep experimentally infected by BSE with a brain homogenate from a BSE-affected cow from France (*21*), and the other (CH41x76) from a goat that had been intracerebrally inoculated with a brain homogenate from cattle BSE from Britain (*28*).

Two experimental scrapie sources in sheep (provided by N. Hunter, Institute for Animal Health, Edinburgh, UK) were also studied. These were the SSBP/1 scrapie isolate, experimentally maintained by serial passages in sheep (*29*), and CH1641 that was derived from a Cheviot sheep and then maintained by serial passages in sheep (*26*).

Natural TSE isolates in small ruminants (Table) were mainly obtained through an active surveillance program in France. In operation since 2002, this program involves the random selection and testing of samples from rendering plants and slaughterhouses (*30*). Some samples originated from the passive surveillance program, which involves collecting samples from animals showing suspected clinical signs of the disease. Brain stems from all selected animals are subjected to a rapid test, and all reactive samples are then retested in the National Reference Laboratory by confirmatory methods based on Western blot (*31*). When a sample is confirmed as TSE positive, it is classified as similar, or not similar, to Nor98 (atypical scrapie), according to the PrP^res^ molecular profile, which in atypical scrapie shows 5 major bands (*31*). Samples that are not similar to Nor98 (classical TSE), i.e, showing a 3-band pattern between 19–30 kDa, are then studied by using a Western blot discriminatory method to identify possible similarities with BSE (*11*).

**Table:** Results of TSE diagnostic tests and molecular characterization for sheep and goats, France, 2002–2009*

Year	Species	No. animals tested by rapid tests	Confirmed TSE cases	Nor98-like isolates	Molecular characterization			
					Not analyzed	Not defined	Scrapie	BSE-like
2002	Ovine	68,580	153	15	0	16	122	0
Caprine	27,087	14	1	0	3	9	1
2003	Ovine	63,207	117	28	0	7	81	1
	Caprine	23,161	10	2	0	2	6	0
2004	Ovine	24,639	58	7	3	0	48	0
	Caprine	5,730	3	0	0	0	3	0
2005†	Ovine	34,290	74	9	19	1	44	1
	Caprine	148,338	16	4	2	1	9	0
2006‡	Ovine	492,023	382	182	1	6	190	3
	Caprine	165,606	10	1	0	1	8	0
2007§	Ovine	327,894	264	173	1	4	86	0
	Caprine	183,498	7	4	0	2	1	0
2008¶	Ovine	86,269	71	45	0	0	26	0
	Caprine	79.966	12	8	0	0	3	1
2009	Ovine	55,163	34	22	0	1	11	0
	Caprine	52,248	6	3	0	0	3	0
Total	Ovine	1,152,065	1,153	481	24	35	608	5
	Caprine	685,634	78	23	2	9	42	2

*TSE, transmissible spongiform encephalopathy; BSE, bovine spongiform encephalopathy. †January 2005, beginning of exhaustive testing of goats in abattoir and rendering plant. ‡First quarter 2006, beginning of exhaustive testing of sheep in abattoir and rendering plant. §January 2007, end of exhaustive testing of sheep in abattoir. ¶February 2008, end of exhaustive testing of goats in abattoir and sheep in rendering plant.

### Western Blot

All confirmed classical TSE (non–Nor98-like) cases were analyzed to determine whether the PrP^res^ looked similar to that found in bovine BSE (i.e., showed a lower molecular mass than that found in bovine BSE) or, on the contrary, was similar as in most scrapie cases. PrP^res^ extracts were obtained from animal brains by using Bio-Rad protocol (the TeSeE Western Blot kit, ref: 355 1169; Bio-Rad, Marnes-la-Coquette, France), which is used to confirm suspected TSE-positive samples and includes PK digestion and rapid protein precipitation steps. Denatured samples were then loaded on two 15% bis-polyacrylamide gels with the same gel plan. After electrophoresis (200 volts for 80 min), the proteins were transferred onto a nitrocellulose membrane (R-Biopharm, St. Didier au Mont d’Or, France). Blocking was performed for 1 h in 5% (wt/vol) milk powder in phosphate-buffered saline (PBS) containing 0.1 % (vol/vol) Tween-20 (PBS-T) for the membrane to be treated with monoclonal antibody Bar233 (1/5000) (144-FGNDYEDRYYRE-155 ovine PrP sequence) (provided by J. Grassi, C.E.A., Saclay, France) or in 3% (wt/vol) bovine serum albumin solution (Sigma, St. Quentin-Fallavier, France) in PBS-T for the membrane to be treated with monoclonal antibody P4 (0.2 mg/mL) (93-WGQGGSH-99 ovine PrPsequence) (R-Biopharm).

Both antibodies were incubated on the membranes for 30 min at room temperature. The membranes were then washed for 20 min in PBS-T and incubated with a solution of streptavidine-peroxidase–conjugated antimouse immunoglobulin (Southern Biotech distributed by Clinisciences, Montrouge, France) in PBS-T for 20 min at room temperature. The membranes were then washed for 30 min in PBS-T and for 5 min in PBS before detection by use of enhanced chemiluminescent substrate (Amersham Biosciences, Orsay, France). The signals were identified on autoradiographic films (Amersham) after a 3-min exposure. Quantitative studies were performed by using Quantity One software (Bio-Rad), and the apparent molecular masses were determined by comparing the positions of the PrP^res^ bands with a biotinylated marker (B2787) (Sigma). All samples were compared, during molecular characterization of field isolates, by expressing the molecular mass of the unglycosylated band in terms of differential molecular mass (dmm). The dmm corresponds to the difference measured between the test sample and the control cattle BSE sample always loaded beside it.

The possible presence of additional C-terminal PrP^res^ products (PrP^res^ #2) (*17*) was detected and quantified by deglycosylation by using peptide N-glycosidase F (PNGase) (kit P07043; BioLabs distributed by Ozyme, Saint-Quentin-en-Yvelines, France) as described (*17*). The deglycosylated PrP^res^ was detected with SAF84 (0.6 mg/mL) (167-RPVDQY-172 ovine PrP sequence) (SPI-Bio, Montigny le Bretonneux, France) mouse monoclonal antibody. The respective proportions of ≈14- and ≈19-kDa bands, observed after PNGase deglycosylation, were quantified by using Quantity One software (Bio-Rad).

## Results

### Active Surveillance Findings during 2002–2009

Since active surveillance of TSEs in small ruminants began in France in 2002, a total of 1,231 small ruminant (1,153 sheep and 78 goats) samples have been confirmed as TSE-positive by Western blot using Sha 31 antibody; >1.7 million animals have been tested by rapid tests (1,152,065 sheep and 685,634 goats) (Table). Nearly half of the positive isolates (504) have been identified as Nor98 scrapie isolates, on the basis of detection of 5 major bands, including a prominent ≈10–12 kDa band detected by Western blot (*32*). After confirmatory Western blot, insufficient quantities of brain tissue were available for Western blot discriminatory testing in 26 samples, which are shown in the Table as samples not analyzed.

The other classical TSE cases, i.e., non–Nor98 cases (648 sheep and 53 goats), which typically showed a 3-band pattern between 19–30 kDa, have been further characterized by applying a discriminatory Western blot method, described as the Agence française de sécurité sanitaire des aliments (French Food Safety Agency) discriminatory method in the Technical Handbook for National Reference Laboratories (*33*). This method enables rapid identification of PrP^res^ patterns similar to those found in experimental ovine BSE and is essentially based on comparison of the PrP^res^ molecular mass with that of cattle BSE, and comparative labeling with 2 antibodies, P4 and Bar233, against either the N terminal end or core part of the PrP^res^ protein, respectively.

### Molecular Characterization of Experimental Isolates

**Figure 1:**
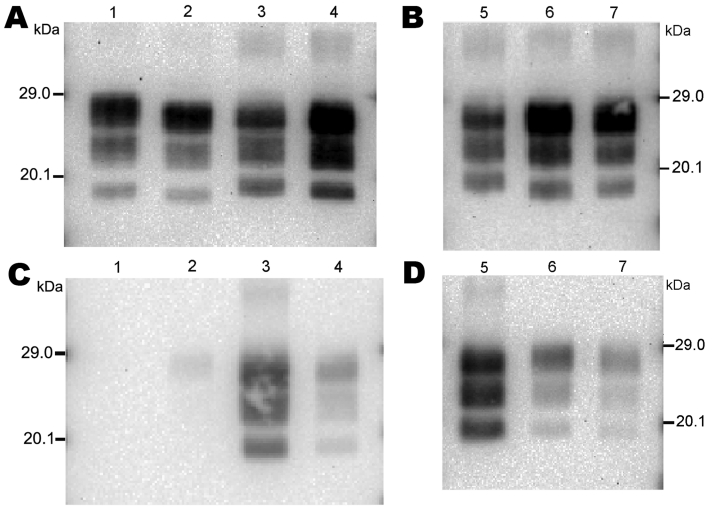
Immunoblots obtained for reference brain samples by discriminatory Western blot method. The first membrane (A, B) was probed with Bar233 antibody. The second membrane (C, D) was probed with monoclonal antibody P4. The 2 immunoblots were loaded with a natural classical bovine spongiform encephalopathy (BSE) isolate (lane 1); an isolate from a sheep experimentally infected with classical BSE 4 (SB1, lanes 2, 6); 2 sheep-passaged scrapie isolates (SSBP/1, lanes 3, 5; CH1641, lane 4); and an isolate from a goat experimentally infected with classical BSE (CH41x76, lane 7).

**Figure 2:**
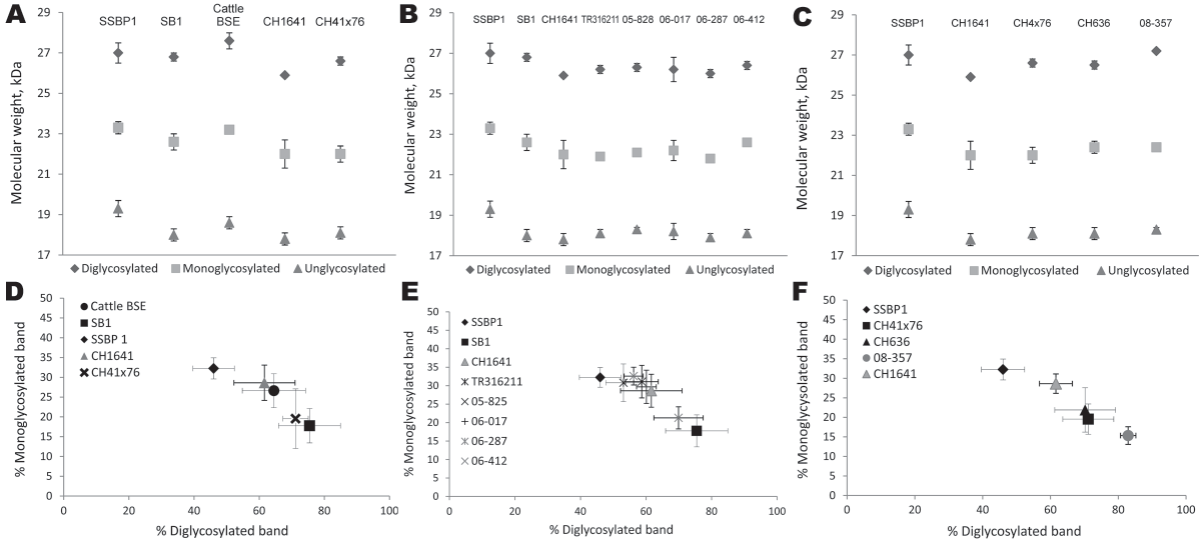
Molecular mass obtained for the di-, mono-, and unglycosylated protein bands (A–C) and the glycoform proportions (D–F) between the diglycosylated band and the monoglycosylated band of the protease-resistant prion protein of the reference transmissible spongiform encephalopathies isolates (A, D), CH1641-like isolates in sheep (B, E), and unusual isolates in goats (C, F). Results were obtained from immunoblots detected by Bar233 antibody. BSE, bovine spongiform encephalopathy.

We first analyzed 4 reference experimental isolates in small ruminants: a sheep and a goat with BSE, 2 sheep infected with SSBP/1 or CH1641 scrapie sources, and a bovid with classical BSE. The mean molecular masses of the diglycosylated (H), monoglycosylated (L), and unglycosylated (U) PrP^res^ bands (using Bar233 antibody) and the P4/Bar233 differential labeling, as well as the proportions of glycoforms, are shown in Figure 1 and in Figure 2, panel A. These analyses show the lower molecular mass of the unglycosylated PrP^res^ in BSE in sheep (–0.6 kDa) and 1 goat (–0.5 kDa), and in CH1641 scrapie (–0.8 kDa), compared with the cattle BSE (Figure 1; Figure 2, panel A). In contrast, SSBP/1 showed a higher apparent molecular mass (+0.6 kDa). The molecular masses of the 3 bands obtained for CH1641 (H: –0.8 kDa, L: –0.3 kDa and U: –0.2 kDa) were lower than in BSE in sheep or goats. Differential labeling by Bar233 and P4 antibodies was correlated with the molecular masses of the protease-resistant cores, with high and low Bar233/P4 ratios in SSBP/1 and BSE in sheep, respectively (Figure 1). Like BSE in sheep, CH1641 showed a decreased signal with P4 compared with Bar 233. bovine BSE showed no labeling with P4 antibody.

Comparison of the proportions of diglycosylated and monoglycosylated bands showed the highest levels of diglycosylated PrP^res^ in experimental BSE in sheep (75 ± 9%/18 ± 4%) and goats (71 ± 7%/20 ± 4%), even compared with cattle BSE (64 ± 10%/27 ± 4%) (Figure 2, panel D). On the contrary, SSBP/1 showed much lower levels of diglycosylated PrP^res^ (46 ± 6%/32 ± 3%), whereas CH1641 was close to cattle BSE (62 ± 9%/29 ± 5%).

Experimental BSE in a goat showed the same molecular features as those observed in experimental BSE in sheep. Both molecular masses, P4 immunolabeling and glycoforms proportions, were involved (Figure 1; Figure 2, panels A, D).

### Molecular Characterization of Natural TSE Sheep and Goat Isolates

We then compared the cattle BSE control with the 701 classical samples available for further analyses (648 sheep and 53 goats). Most of the isolates tested (657) were PrP^res^ positive by discriminatory Western blot using Bar233 core antibody and showed a 3-band pattern, whereas the signal was weaker in 44 samples, preventing the identification and characterization of the unglycosylated band.

**Figure 3:**
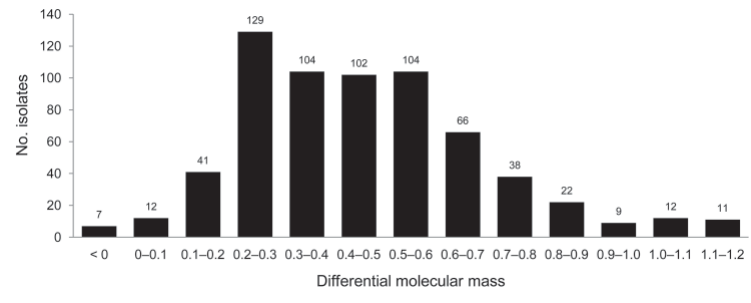
Differences in molecular mass observed between protease-resistant prion protein in cattle bovine spongiform encephalopathy (BSE) and usual transmissible spongiform encephalopathy cases in small ruminants. Differential molecular mass was obtained by subtracting the molecular mass of the unglycosylated band of the cattle BSE control to that of the natural small ruminant isolate from an immunoblot detected by Bar233 antibody.

We chose to express the molecular masses measures as dmm, which corresponds to the difference of molecular masses measured between the tested sample and the control cattle BSE always loaded beside it. Most (650) samples showed a positive dmm (Figure 3) and strong labeling with P4 antibody, as previously described for SSBP/1. However, large variations in molecular mass (1.2 kDa) were observed among these samples, a possible clue pointing to biological diversity among scrapie sources; in contrast, only small variations were observed after repeated measures of a same sample with the Western blot method, as shown by the small standard deviations during repeated analysis of reference samples (Figure 2). A minority of the samples (*12*) showed similar molecular masses to bovine BSE (dmm <0.1 kDa difference). Stronger P4 labeling compared with Bar 233 antibody was observed in all these samples and in the 44 (not defined) samples for which the dmm could not be measured with Bar233 antibody because quantities of extracted PrPres were too low.

A few samples (5 sheep and 2 goats) showed a lower PrP^res^ molecular mass than the bovine BSE control, i.e., a negative dmm (black bar in Figure 3), combined with poor labeling by P4 antibody, as described for BSE in small ruminants and CH1641 scrapie. These samples were suspected to represent BSE in small ruminants and were thus further characterized by repeated Western blot analyses in comparison with experimental controls.

### Molecular Studies of TSE Isolates with Low Molecular Mass PrP^res^

These experiments allowed repeated measures of PrP^res^ molecular masses and P4/Bar233 differential labeling and estimation of the proportions of PrP^res^ glycoforms. Western blot analyses were also performed with the C-terminal SAF84 antibody to identify possible additional C-terminal PrP^res^ cleavage products, as described for the CH1641 experimental isolate (*17*).

**Figure 4:**
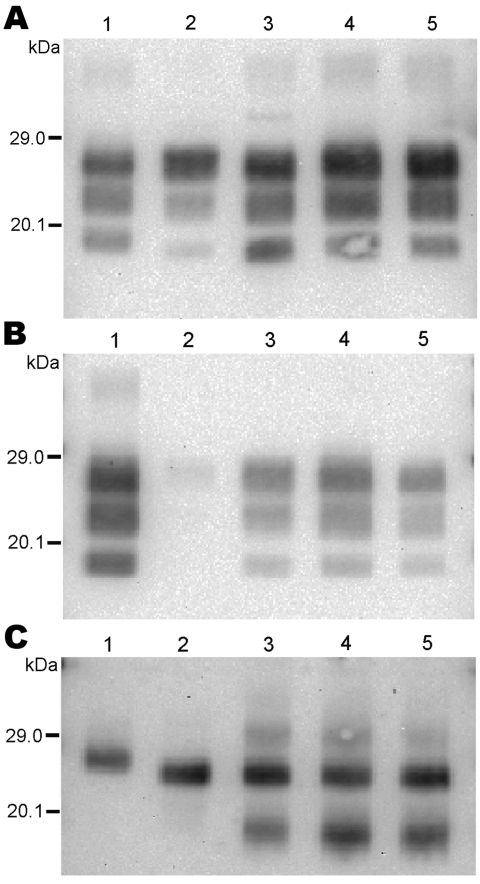
Western blot analysis of protease-resistant prion protein in 2 CH1641-like sheep isolates (06-017, lane 3; 06-287, lane 4) detected by Bar233 (A), P4 (B), and SAF84 (C) antibodies. These samples were compared with 2 sheep-passaged scrapie isolates (SSBP/1, lane 1; CH1641, lane 5) and an isolate from a sheep experimentally infected with classical spongiform encephalopathy (SB1, lane 2). Samples in panel C were deglycosylated with peptide N-glycosidase F before Western blot analysis.

The 3 PrP^res^ glycoforms of the 5 sheep isolates showed similar molecular masses close to BSE in sheep or CH1641 scrapie (Figure 2, panel B; Figure 4, panel A). These similarities of PrPres migration were associated with intermediate labeling with P4 but nevertheless varied considerably (up to 3×) according to the isolate (data not shown). Analyses of the glycoform proportions showed lower proportions of the diglycosylated band, compared with BSE in sheep, and also similarity to CH1641 in this respect (Figure 2, panel E). All 5 isolates showed an additional ≈14 kDa PrP^res^ band, after use of the SAF84 antibody (Figure 4, panel C), that in 4 isolates represented 30 ± 7% to 34 ± 7% of the total signal after repeated analyses and PNGase deglycosylation. Among these sheep isolates, the 06-412 sample showed higher molecular masses and proportion of diglycosylated band. Nevertheless, the 5 natural isolates thus appeared similar to the CH1641 experimental scrapie isolate.

**Figure 5:**
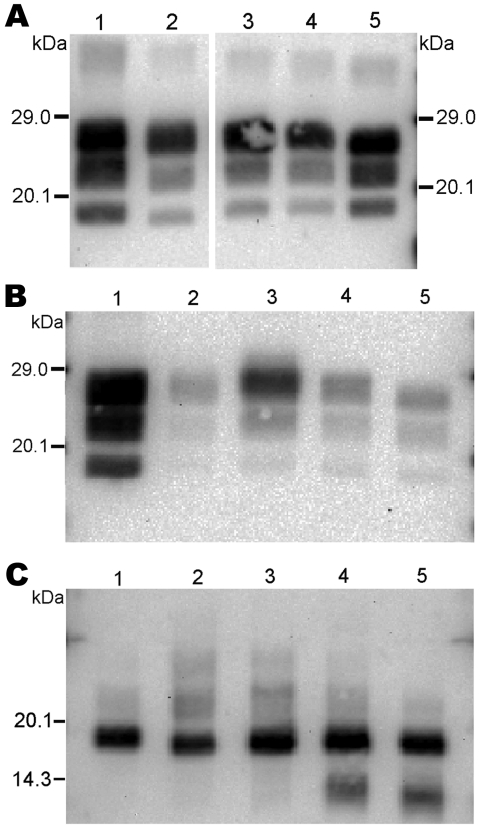
Western blot analysis of protease-resistant prion protein in 2 goat isolates (CH636, lane 3; 08-357, lane 4) detected by Bar233 (A), P4 (B), and SAF84 (C) antibodies. These samples were compared with an isolate from a goat naturally infected with scrapie (lane 1); an isolate from a goat experimentally infected with classical BSE (CH41x76, lane 2); and a sheep-passaged scrapie isolate (CH1641, lane 5). Samples in panel C were deglycosylated with peptide N-glycosidase F before Western blot analysis.

For the 2 goat samples with low molecular mass PrP^res^, the molecular characteristics of the CH636 isolate were indistinguishable from BSE in goat (CH41x76), with regard to PrP^res^ molecular masses (Figure 5, panel A) and the respective proportions of the diglycosylated/monoglycosylated bands (70 ± 9%/22 ± 6% and 71 ± 8%/20 ± 4%, respectively) (Figure 2, panel F) when Bar233 antibody was used, and reduced labeling by P4 antibody compared with Bar233 (Figure 5, panel B). A similar pattern was observed with the other goat sample 08-357, but the levels of the diglycosylated band were even higher (83 ± 2%). However, Western blot analyses with SAF84 antibody showed that these 2 goat samples clearly differed and had an additional ≈14 kDa PrP^res^ band only in the 08-357 isolate, as in the CH1641 experimental scrapie isolate. This ≈14 kDa PrP^res^ band was represented in the same proportions as in the CH1641-like sheep isolates. This sample could not be clearly distinguished from BSE in goat according to other molecular criteria.

## Discussion

We investigated the PrP^res^ molecular features of one of the largest series of natural TSE isolates from sheep and goats analyzed so far in Europe. Approximately 1.7 million small ruminants were subjected to a rapid test; among these, 1,153 sheep and 78 goats originating from 992 flocks were confirmed as TSE-positive on the basis of identification of PrP^res^ in the brain stem. Another large study using 1,247 sheep originating from 450 flocks in Great Britain has been reported (*24*). Similar studies have been conducted in other European countries such as Germany (*34*), the Netherlands (*14**,*) and Italy (*23*), but fewer TSE-positive animals were reported. In our series, animals with classical cases represented 53% of the TSE-affected animals.

The molecular features of most of these isolates (99%) were comparable to those previously described for most scrapie cases, in studies in Europe or France (*21*). In all of these cases, PrP^res^ from sheep and goats showed clearly distinct features from BSE, based on the identification of a higher molecular mass of unglycosylated PrP^res^, associated with strong labeling by the P4 antibody that recognizes the N terminal end of the protein. However, our observation of large individual variations in this PrP^res^ molecular mass implies that a possible relationship with the biologic diversity of TSE agents, which has been described after transmission of scrapie to inbred wild-type mice (*35*), would be questionable. Only a few animals (5 sheep and 2 goats) in this large series of TSE-affected animals showed molecular characteristics that, in comparison with the usual features of scrapie, could be consistent with the known BSE signature in small ruminants. These samples represented all the suspected isolates that were identified by official surveillance in France during 2002–2009.

An essential molecular criterion defined from the observation of PrP^res^ BSE compared with scrapie was the low molecular mass of the unglycosylated band in PrPres BSE, associated with a decreased PrP^res^ signal lower with an N terminal antibody than with a core antibody (*13**,**22**,**36*). After identification of these molecular features in a few small ruminants, only 1 (CH636) of the 2 cases here described in goats, identified as TSE positive in 2002, has been shown to be infected by the BSE agent after bioassays in mice (*37*). Another goat in the United Kingdom identified by an immunohistochemical discriminatory method as TSE-positive in 1990 showed characteristics that were indistinguishable from BSE (*18*). These results clearly indicate that in a situation characterized by a decrease in the number of cases in cattle in all countries in Europe, the possibility of finding BSE in small ruminants is now remote.

The other unusual isolates showed molecular characteristics that were partly similar, not only to BSE in small ruminants with a low molecular mass of PrP^res^ and faint labeling with P4 antibody, but also to the CH1641 experimental scrapie isolate. However, detailed immunohistochemical investigations of CH1641 showed subtle differences in the cleavage site of the protein compared with BSE in sheep (*38*). As previously described, after transmission in ovine transgenic mice (*17*), the slightly lower PrP^res^ molecular mass in CH1641-like isolates, as recognized in the CH1641 experimental isolate (*13*),was confirmed by Western blot, at least in sheep (Figure 2, panel B). However, these differences in molecular mass are more easily identified on the diglycosylated band. Compared with BSE in small ruminants, lower proportions of this diglycosylated band were found in sheep, whereas the 08-357 goat sample showed very high levels of this diglycosylated band, which would be consistent with BSE. Experimental transmissions of BSE in sheep have shown that, to a certain extent, the PrP^res^ molecular features could be influenced by different factors, such as serial passages in sheep (*39*) or sheep genotypes (*36*), although slight variability did not compromise the discrimination with scrapie. Furthermore, all these CH1641-like natural isolates in sheep and goats clearly differed from BSE by the presence of an additional, C-terminally cleaved, PrP^res^ product specifically recognized by a C-terminal antibody (SAF84), as previously described for the CH1641 experimental scrapie isolate (*17*). Baron et al. described bioassays of 3 of 20 CH1641-like sheep isolates (*17*), which are also being conducted for the other isolates.

These 6 CH1641-like isolates were identified among 1,153 sheep and 78 goats with confirmed TSE, and the goat case represents, to our knowledge, a spreading of the known species range for natural CH1641 infection. At least in sheep, for which 4.34 cases per million sheep tested were identified in this study, the frequency of CH1641-like scrapie was notably higher compared with other rare TSEs in ruminants such as atypical BSEs, which showed a frequency of 0.76 per million cattle tested during 2001–2007 (*40*). Thus, large-scale testing of animals would be required to identify these rare TSE isolates. Similar isolates were only identified in sheep in the other large series reported from 450 flocks in Great Britain (2 cases in 1 flock) (*23*) and in a previous study of 214 TSE-infected sheep in France (2 cases in 1 flock) (*27*). However, an underestimation of the frequency of such cases cannot be fully excluded. PrP^res^ features are assessed by analyzing a single homogenate prepared from a brain fragment from the animal. Stack et al. described a case in sheep that appeared as CH1641-like after repeated Western blot analysis of a brain stem sample, whereas previous analysis of the caudal medulla at the time of submission had shown the usual scrapie profile (*24*). Immunohistochemical testing of 2 CH1641-like cases in sheep showed, that unlike BSE, PrP^d^ could be clearly identified by using P4 antibody in some of the brain stem nuclei and in lymphoid tissues (*27*). Finally, on the basis of identification of low levels of C-terminal PrP^res^ product in ovine transgenic mice infected with usual scrapie isolates, we hypothesized that a CH1641-like component might be present as a minor component in these scrapie cases that showed usual molecular features (*17*). All these data raise the question of the existence of possible mixtures of TSE agents in these particular CH1641-like isolates.

## References

[1] Collinge J, Sidle KC, Meads J, Ironside J, Hill AF. Molecular analysis of prion strain variation and the aetiology of ‘new variant’ CJD. Nature. 1996;383:685–90. 10.1038/383685a0 8878476

[2] Hill AF, Desbruslais M, Joiner S, Sidle KC, Gowland I, Collinge J, The same prion strain causes vCJD and BSE. Nature. 1997;389:448–50, 526. 10.1038/38925 9333232

[3] Prusiner SB. Prions. Proc Natl Acad Sci U S A. 1998;95:13363–83. 10.1073/pnas.95.23.13363 9811807 PMC33918

[4] Caughey B, Raymond GJ. The scrapie-associated form of PrP is made from a cell surface precursor that is both protease- and phospholipase-sensitive. J Biol Chem. 1991;266:18217–23. 1680859

[5] Riek R, Hornemann S, Wider G, Glockshuber R, Wuthrich K. NMR characterization of the full-length recombinant murine prion protein, mPrP(23–231). FEBS Lett. 1997;413:282–8. 10.1016/S0014-5793(97)00920-4 9280298

[6] Riesner D. Biochemistry and structure of PrP(C) and PrP(Sc). Br Med Bull. 2003;66:21–33. 10.1093/bmb/66.1.21 14522846

[7] Gavier-Widen D, Stack MJ, Baron T, Balachandran A, Simmons M. Diagnosis of transmissible spongiform encephalopathies in animals: a review. J Vet Diagn Invest. 2005;17:509–27. 10.1177/104063870501700601 16475509

[8] Bruce M, Chree A, McConnell I, Foster J, Pearson G, Fraser H. Transmission of bovine spongiform encephalopathy and scrapie to mice: strain variation and the species barrier. Philos Trans R Soc Lond B Biol Sci. 1994;343:405–11. 10.1098/rstb.1994.0036 7913758

[9] Foster JD, Hope J, Fraser H. Transmission of bovine spongiform encephalopathy to sheep and goats. Vet Rec. 1993;133:339–41. 10.1136/vr.133.14.339 8236676

[10] Opinion of the Scientific Steering Committee of the European Commission on Hypotheses on the Origin and Transmission of BSE. European Commission, adopted 29–30 Nov 2001. http://ec.europa.eu/food/fs/sc/ssc/out236_en.pdf

[11] Opinion of the Scientific Steering Committee of the European Commission on Strategy to Investigate the Possible Presence of BSE in Sheep. European Commission, adopted on 4–5 Apr 2002. http://ec.europa.eu/food/fs/sc/ssc/out281_en.pdf

[12] Hill AF, Sidle KC, Joiner S, Keyes P, Martin TC, Dawson M, Molecular screening of sheep for bovine spongiform encephalopathy. Neurosci Lett. 1998;255:159–62. 10.1016/S0304-3940(98)00736-8 9832197

[13] Stack MJ, Chaplin MJ, Clark J. Differentiation of prion protein glycoforms from naturally occurring sheep scrapie, sheep-passaged scrapie strains (CH1641 and SSBP1), bovine spongiform encephalopathy (BSE) cases, and Romney and Cheviot breed sheep experimentally inoculated with BSE using two monoclonal antibodies. Acta Neuropathol. 2002;104:279–86. 12172914 10.1007/s00401-002-0556-2

[14] Thuring CM, Erkens JH, Jacobs JG, Bossers A, Van Keulen LJ, Garssen GJ, Discrimination between scrapie and bovine spongiform encephalopathy in sheep by molecular size, immunoreactivity, and glycoprofile of prion protein. J Clin Microbiol. 2004;42:972–80. 10.1128/JCM.42.3.972-980.2004 15004040 PMC356877

[15] Notari S, Strammiello R, Capellari S, Giese A, Cescatti M, Grassi J, Characterization of truncated forms of abnormal prion protein in Creutzfeldt-Jakob disease. J Biol Chem. 2008;283:30557–65. 10.1074/jbc.M801877200 18753138 PMC2662149

[16] Biacabe AG, Jacobs JG, Bencsik A, Langeveld JP, Baron TG. H-type bovine spongiform encephalopathy: complex molecular features and similarities with human prion diseases. Prion. 2007;1:61–8. 10.4161/pri.1.1.3828 19164888 PMC2633710

[17] Baron T, Bencsik A, Vulin J, Biacabe AG, Morignat E, Verchere J, A C-terminal protease-resistant prion fragment distinguishes ovine “CH1641-like” scrapie from bovine classical and L-Type BSE in ovine transgenic mice. PLoS Pathog. 2008;4:e1000137. 10.1371/journal.ppat.1000137 18769714 PMC2516186

[18] Jeffrey M, Martin S, Gonzalez L, Foster J, Langeveld JP, van Zijderveld FG, Immunohistochemical features of PrP(d) accumulation in natural and experimental goat transmissible spongiform encephalopathies. J Comp Pathol. 2006;134:171–81. 10.1016/j.jcpa.2005.10.003 16542672

[19] Simon S, Nugier J, Morel N, Boutal H, Creminon C, Benestad SL, Rapid typing of transmissible spongiform encephalopathy strains with differential ELISA. Emerg Infect Dis. 2008;14:608–16. 10.3201/eid1404.071134 18394279 PMC2570920

[20] Baron TG, Madec JY, Calavas D. Similar signature of the prion protein in natural sheep scrapie and bovine spongiform encephalopathy-linked diseases. J Clin Microbiol. 1999;37:3701–4. 10523578 10.1128/jcm.37.11.3701-3704.1999 PMC85730

[21] Baron TG, Madec JY, Calavas D, Richard Y, Barillet F. Comparison of French natural scrapie isolates with bovine spongiform encephalopathy and experimental scrapie infected sheep. Neurosci Lett. 2000;284:175–8. 10.1016/S0304-3940(00)01047-8 10773427

[22] Baron TG, Biacabe AG. Molecular analysis of the abnormal prion protein during coinfection of mice by bovine spongiform encephalopathy and a scrapie agent. J Virol. 2001;75:107–14. 10.1128/JVI.75.1.107-114.2001 11119579 PMC113903

[23] Nonno R, Esposito E, Vaccari G, Conte M, Marcon S, Di Bari M, Molecular analysis of cases of Italian sheep scrapie and comparison with cases of bovine spongiform encephalopathy (BSE) and experimental BSE in sheep. J Clin Microbiol. 2003;41:4127–33. 10.1128/JCM.41.9.4127-4133.2003 12958236 PMC193806

[24] Stack M, Jeffrey M, Gubbins S, Grimmer S, Gonzalez L, Martin S, Monitoring for bovine spongiform encephalopathy in sheep in Great Britain, 1998–2004. J Gen Virol. 2006;87:2099–107. 10.1099/vir.0.81254-0 16760414

[25] Hope J, Wood SC, Birkett CR, Chong A, Bruce ME, Cairns D, Molecular analysis of ovine prion protein identifies similarities between BSE and an experimental isolate of natural scrapie, CH1641. J Gen Virol. 1999;80:1–4. 9934675 10.1099/0022-1317-80-1-1

[26] Foster JD, Dickinson AG. The unusual properties of CH1641, a sheep-passaged isolate of scrapie. Vet Rec. 1988;123:5–8. 10.1136/vr.123.1.5 3140468

[27] Lezmi S, Martin S, Simon S, Comoy E, Bencsik A, Deslys JP, Comparative molecular analysis of the abnormal prion protein in field scrapie cases and experimental bovine spongiform encephalopathy in sheep by use of Western blotting and immunohistochemical methods. J Virol. 2004;78:3654–62. 10.1128/JVI.78.7.3654-3662.2004 15016886 PMC371064

[28] Foster J, Goldmann W, Parnham D, Chong A, Hunter N. Partial dissociation of PrP(Sc) deposition and vacuolation in the brains of scrapie and BSE experimentally affected goats. J Gen Virol. 2001;82:267–73. 11125179 10.1099/0022-1317-82-1-267

[29] Hunter N, Foster JD, Dickinson AG, Hope J. Linkage of the gene for the scrapie-associated fibril protein (PrP) to the *Sip* gene in Cheviot sheep. Vet Rec. 1989;124:364–6. 10.1136/vr.124.14.364 2566212

[30] Morignat E, Cazeau G, Biacabe AG, Vinard JL, Bencsik A, Madec JY, Estimates of the prevalence of transmissible spongiform encephalopathies in sheep and goats in France in 2002. Vet Rec. 2006;158:683–7. 10.1136/vr.158.20.683 16714431

[31] Arsac JN, Biacabe AG, Nicollo J, Bencsik A, Baron T. Biochemical identification of bovine spongiform encephalopathies in cattle. Acta Neuropathol. 2007;114:509–16. 10.1007/s00401-007-0270-1 17668226

[32] Arsac JN, Andreoletti O, Bilheude JM, Lacroux C, Benestad SL, Baron T. Similar biochemical signatures and prion protein genotypes in atypical scrapie and Nor98 cases, France and Norway. Emerg Infect Dis. 2007;13:58–65. 10.3201/eid1301.060393 17370516 PMC2725815

[33] Community Reference Laboratory of the European Union. Discriminatory testing handbook. TSE strain characterisation in small ruminants—a technical handbook for national reference laboratories in the EU. Version 3. 2009 March.

[34] Gretzschel A, Buschmann A, Eiden M, Ziegler U, Luhken G, Erhardt G, Strain typing of German transmissible spongiform encephalopathies field cases in small ruminants by biochemical methods. J Vet Med B Infect Dis Vet Public Health. 2005;52:55–63. 10.1111/j.1439-0450.2005.00827.x 15752263

[35] Bruce ME, Boyle A, Cousens S, McConnell I, Foster J, Goldmann W, Strain characterization of natural sheep scrapie and comparison with BSE. J Gen Virol. 2002;83:695–704. 11842264 10.1099/0022-1317-83-3-695

[36] Ronzon F, Bencsik A, Lezmi S, Vulin J, Kodjo A, Baron T. BSE inoculation to prion diseases–resistant sheep reveals tricky silent carriers. Biochem Biophys Res Commun. 2006;350:872–7. 10.1016/j.bbrc.2006.09.137 17049491

[37] Eloit M, Adjou K, Coulpier M, Fontaine JJ, Hamel R, Lilin T, BSE agent signatures in a goat. Vet Rec. 2005;156:523–4. 15833975 10.1136/vr.156.16.523-b

[38] Jeffrey M, Gonzalez L, Chong A, Foster J, Goldmann W, Hunter N, Ovine infection with the agents of scrapie (CH1641 isolate) and bovine spongiform encephalopathy: immunochemical similarities can be resolved by immunohistochemistry. J Comp Pathol. 2006;134:17–29. 10.1016/j.jcpa.2005.06.005 16324707

[39] Stack M, Gonzalez L, Jeffrey M, Martin S, Macaldowie C, Chaplin M, Three serial passages of bovine spongiform encephalopathy in sheep do not significantly affect discriminatory test results. J Gen Virol. 2009;90:764–8. 10.1099/vir.0.005983-0 19218224

[40] Biacabe AG, Morignat E, Vulin J, Calavas D, Baron TG. Atypical bovine spongiform encephalopathies, France, 2001–2007. Emerg Infect Dis. 2008;14:298–300. 10.3201/eid1402.071141 18258124 PMC2600212

